# Cryptic Methane-Cycling by Methanogens During Multi-Year Incubation of Estuarine Sediment

**DOI:** 10.3389/fmicb.2022.847563

**Published:** 2022-03-17

**Authors:** Richard T. Kevorkian, Katie Sipes, Rachel Winstead, Raegan Paul, Karen G. Lloyd

**Affiliations:** Department of Microbiology, University of Tennessee, Knoxville, TN, United States

**Keywords:** methane, methanogen, marine sediment, sulfate reducer, anaerobic oxidation of methane, sulfate methane transition zone

## Abstract

As marine sediments are buried, microbial communities transition from sulfate-reduction to methane-production after sulfate is depleted. When this biogenic methane diffuses into the overlying sulfate-rich sediments, it forms a sulfate-methane transition zone (SMTZ) because sulfate reducers deplete hydrogen concentrations and make hydrogenotrophic methanogenesis exergonic in the reverse direction, a process called the anaerobic oxidation of methane (AOM). Microbial participation in these processes is often inferred from geochemistry, genes, and gene expression changes with sediment depth, using sedimentation rates to convert depth to time. Less is known about how natural sediments transition through these geochemical states transition in real-time. We examined 16S rRNA gene amplicon libraries and metatranscriptomes in microcosms of anoxic sediment from the White Oak River estuary, NC, with three destructively sampled replicates with methane added (586-day incubations) and three re-sampled un-amended replicates (895-day incubations). Sulfate dropped to a low value (∼0.3 mM) on similar days for both experiments (312 and 320 days, respectively), followed by a peak in hydrogen, intermittent increases in methane-cycling archaea starting on days 375 and 362 (mostly *Methanolinea* spp. and *Methanosaeta* spp., and *Methanococcoides* sp. ANME-3), and a methane peak 1 month later. However, methane δ^13^C values only show net methanogenesis 6 months after methane-cycling archaea increase and 4 months after the methane peak, when sulfate is consistently below 0.1 mM and hydrogen increases to a stable 0.61 ± 0.13 nM (days 553–586, *n* = 9). Sulfate-reducing bacteria (mostly *Desulfatiglans* spp. and *Desulfosarcina* sp. SEEP-SRB1) increase in relative abundance only during this period of net methane production, suggesting syntrophy with methanogens in the absence of sulfate. The transition from sulfate reduction to methane production in marine sediments occurs through a prolonged period of methane-cycling by methanogens at low sulfate concentrations, and steady growth of sulfate reducers along with methanogens after sulfate is depleted.

## Introduction

Marine sediments produce ∼85 Tg/yr of the greenhouse gas methane, making them the third largest producer of methane on Earth (D’Hondt et al., 2002; Reeburgh, 2007; Bange et al., 2009; Bradley et al., 2020). However, only ∼10 Tg/yr of this methane is emitted to the atmosphere, well behind emissions from rice paddies, wetlands, animals, landfills, biomass burning, and commercial gas production (Reeburgh, 2007). This discrepancy is because most of the methane produced in marine sediments is consumed by sulfate-dependent anaerobic oxidation of methane (AOM) before it has a chance to escape to the atmosphere or overlying water. Most methanogenesis is microbial and derives its substrates from the products of organic matter fermentation below the depth of sulfate depletion (Reeve et al., 1997). Hydrogenotrophic methanogens use hydrogen and carbon dioxide, acetoclastic methanogens use acetate, and methylotrophic methanogens use methylated compounds (Reeve et al., 1997). This methane diffuses up through the sulfate/methane transition zone (SMTZ) where it is oxidized through reverse hydrogenotrophic methanogenesis, since sulfate reducers keep hydrogen concentrations low enough to make this process exergonic in the reverse direction (Hoehler et al., 1994, 1998; Reeburgh, 2007; Egger et al., 2018). This is distinct from the direct electron transfer and nanowire connections that have been observed for methane seeps operating at much higher rates of AOM and in the presence of high hydrogen concentrations (Alperin and Hoehler, 2010; Mcglynn et al., 2015; Wegener et al., 2015).

Much of what is known about the microbes that drive the transition from sulfate reduction to methanogenesis in marine sediments has been determined by coupling microbiological and geochemical measurements across sediment core depth gradients [e.g., (Leloup et al., 2009; Lloyd et al., 2011; Kevorkian et al., 2021)]. When rates of sediment accumulation and porewater advection are known, reaction transport models can be used to calculate microbial rates of methanogenesis, sulfate reduction, and AOM (Alperin et al., 1988). However, such studies are complicated by the fact that both AOM and methanogenesis occur simultaneously with sulfate reduction in the SMTZ, when measured by turnover of trace amounts of radioactively labeled substrates in sediments in Cape Lookout Bight, NC (Hoehler et al., 1994), Aarhus Bay, Denmark (Beulig et al., 2019), and Skagerrak, Denmark (Parkes et al., 2007; Knab et al., 2008). This methane-cycling is “cryptic” because methanogenesis and AOM both occur at the same sediment depth layers, even though the change in methane concentrations through the depth layers indicates net AOM within the SMTZ and net methanogenesis below it. Most near-equilibrium enzymatic processes will express some back reaction, like a leaky valve. However, the methanogenesis rates that occur simultaneously with AOM correlate with sulfate reduction rates and even exceed them at very low sulfate concentrations, making it unlikely that the methane-cycling is due to a back reaction of AOM (Beulig et al., 2019).

Some evidence supports the possibility that microbes can conserve energy from methane-cycling. Hydrogenotrophic methanogenesis can be reversed to AOM at low hydrogen concentrations, although it does not provide sufficient energy to sustain actively growing laboratory cultures (Valentine et al., 2000). Natural marine sediment communities, however, operate at orders of magnitude lower energy yields than laboratory cultures (Hoehler and Jørgensen, 2013), and for such communities, AOM provides sufficient energy (Martens and Berner, 1977; Iversen and Jorgensen, 1985). The ability to reverse between methanogenesis and AOM based on hydrogen concentrations has been observed in enrichments of uncultured ANME-1 archaea (Wegener et al., 2015), a clade that is common in marine sediments (Harrison et al., 2009; Lloyd et al., 2011; Underwood et al., 2016; Beulig et al., 2019; Kevorkian et al., 2021). ANME-1 are dominant and active in both in the SMTZ during net AOM and in the net methanogenic sediments below it, suggesting they catalyze either direction (Lloyd et al., 2011). Furthermore, subpopulations of ANME-1 have a heterogeneous distribution (Kevorkian et al., 2021) and substrate usage (House et al., 2009) within the SMTZ, suggesting that metabolic flexibility during methane-cycling may lead to the observed patchiness of their distribution and cellular δ^13^C biomass values.

Together, these studies suggest that the transition from sulfate reduction to methanogenesis in marine sediments is more complex than a simple cessation of sulfate reduction at the bottom of the SMTZ, with a switch from only AOM above to only methanogenesis below. Long-term incubations on natural marine sediments allow these processes to be measured in real-time, linking microbes to the processes that occur while they grow, thus removing some of the uncertainty of downcore studies (Alperin et al., 1992; Timmers et al., 2015; Kevorkian et al., 2018). If the organic matter is sufficiently labile, natural quantities of sulfate can be consumed in just a few months, followed by net methane production (Alperin et al., 1992; Kevorkian et al., 2018). However, many marine sediments have less labile organic matter than that of the Cape Lookout Bight sediments used in these studies (Martens et al., 1998). Performing long-term incubations with less organic rich sediments may therefore slow the processes down and display these complex transitions in real-time.

We performed laboratory incubations of marine-influenced White Oak River estuary sediments, which are typical of non-methane-emitting methanogenic sediments. Here, all methane is removed via AOM in the SMTZ and the flux of organic matter is 7- to 30-fold lower than Cape Lookout Bight (Martens et al., 1998; Lloyd et al., 2011). We performed 3 replicate 586-day anoxic incubations of the top 3 cm of White Oak River (WOR) estuary sediment, with timepoints destroyed from 54 separate bottles, each of which had a single addition methane added to the headspace on day 44 (incubation WOR 5.17) to attempt to stimulate AOM while sulfate was available. In a separate experiment, we performed 3 replicates 895-day incubations of subsampled and unamended anoxic incubations of the top 3 cm of WOR sediments (incubation WOR 5.16). We tracked both the geochemistry (methane, sulfate, hydrogen for both incubations, and δ^13^C of methane for the WOR 5.17 incubation) and microbial community composition (16S rRNA gene amplicon libraries for both and transcriptomics for WOR 5.16) to observe changes in the microbial community during the transition from sulfate reduction to methanogenesis.

## Materials and Methods

### Sample Collection

Plunger cores were collected in May 2016 (WOR 5.16) and May 2017 (WOR 5.17) (multiple 30cm length cores) at the White Oak River estuary station H (34 44.490’ N, 77 07.44’ W), in 1.5 m water depth. Using a plunger inserted from the bottom, the first three centimeters of sediment from each core was removed and mixed together in a sterile flask. For WOR 5.16, 1.5 L of the slurry was placed in three 2L Erlenmeyer flasks, similar to the method used in Kevorkian et al. (2018) (Supplementary Figure 1A). The flask was stoppered with a butyl stopper fitted with a wide-bore stopcock for sediment subsampling and gas sampling ports. For WOR 5.16, about 100 ml of sediment were autoclaved and incubated alongside the experiments under anoxic conditions as a negative control. For WOR 5.17, 20 ml of the slurry was added to 54 60 ml glass serum vials, plus two more with autoclaved sediments, and three more with autoclaved distilled water (Supplementary Figure 1B). All vials were stoppered with thick butyl rubber stoppers, crimp sealed, and the headspace was gassed with O_2_-scrubbed ultra-high purity N_2_ gas.

### Sampling and Geochemical Measurements

Each of the three WOR5.16 flasks were fitted with a custom butyl rubber stopper with a hole drilled through the center to accommodate a wide bore (6 mm) glass and Teflon stopcock for the removal of samples. Two 18-gauge needles with stainless steel stopcocks were inserted into the stopper as well. Using the luer-lock fitting on the needles, ultra-high purity nitrogen gas (99.999%) that had been scrubbed of oxygen using heated copper fillings was flowed through the bottles using the second needle for the outflow to make the headspace anoxic. Then the flasks were let stand at constant room temperature (21.4°C), which is the temperature of the samples when they were taken, in the dark.

The WOR5.16 incubations were turned over once every month just before sampling. Prior to gas sampling, 2 ml of anoxic N_2_ gas (99.999%) was used to blow the needle clear of sediment. Separate hydrogen and methane gas samples were collected in glass gastight Hamilton syringes using the steel needle ports in the custom stopper. About 32 ml of sediment was removed through the glass and Teflon stopcock using a sterile 60 ml plastic catheter tip syringe. After sampling, 30 ml of oxygen- and hydrogen-scrubbed N_2_ was injected into the bottle to replace the lost volume.

The WOR5.17 incubation bottles were sampled destructively in triplicate on a monthly basis. The crimp seal and butyl stopper were removed after sampling the headspace for hydrogen, methane, and stable carbon isotopes. On day 44, 4 ml methane was added by syringe to the 40 ml headspace, which is 5.11 mM when equilibrated with porewater.

From both incubations, two 15 ml conical centrifuge tubes were filled and capped, one used for porewater analysis and the other frozen at −80°C for later molecular analysis. One ml of sediment was placed in a 2 ml screw cap tube with 3% paraformaldehyde. The 15 ml tube destined for porewater analysis was centrifuged at 5 000 × *g* for 5 min. A syringe was used to remove the supernatant not in contact with the air. The porewater was then filtered using a 0.2 μm syringe filter into 100 μl of 10% HCl to a final volume of 1 ml. Porewater sulfate concentrations were determined by ion chromatography (Dionex, Sunnyvale, CA, United States).

A total of 500 μl of headspace gas was injected into a Peak Performer 1 Reducing Compound Photometer (Peak Laboratories, Mountain View, CA, United States). Premixed hydrogen ppm lab bottles (Airgas, Radnor, PA, United States) were used as standards. Hydrogen was assumed to be equilibrated between headspace and porewater. Methane was determined by injecting 500 μl of gas from the headspace into an evacuated glass bottle to be later analyzed on a gas chromatograph with a flame ionization detector (Agilent, Santa Clara, CA, United States). Methane concentrations were not assumed to be equilibrated with the aqueous phase; therefore, concentrations are presented as headspace partial pressures. The formula for determining methane concentration was peak area of sample multiplied by the volume of the bottle headspace, which was divided by gas constant times temperature, porosity, volume of sediment.

To measure δ^13^C values of methane, 4 ml of headspace from the vial used for methane measurements was removed via syringe and injected into a gas bag containing hydrocarbon free zero gas (Airgas, Radnor, PA, United States). This was then measured on a cavity ring down spectrometer using a small sample introduction module (Picarro, Santa Clara, CA, United States).

### Cell Quantification

Total cell counts were determined by direct epifluorescence microscopy SYBRGold DNA stain (Invitrogen, Carlsbad, CA, United States). Sediments were sonicated at 20% power for 40 s to disaggregates cells from sediments and diluted 40-fold into PBS prior to filtration onto a 0.2 μm polycarbonate filter (Fisher Scientific, Waltham, MA, United States) and mounted onto a slide.

### 16S Ribosomal RNA Gene Amplicons

DNA was extracted from WOR5.16 frozen sediments using the Qiagen Powersoil Total DNA extraction kit. DNA was extracted from WOR5.17 frozen sediments using a protocol modified from Mills et al. (2008). DNA was extracted from each of the three separate bottles that were destroyed at each timepoint. Autoclaved sediment and water blanks were used as negative controls. The V4 region of each DNA extraction was amplified using primers 806r and 515f (Caporaso et al., 2012), as a universal primer pair for Bacteria and Archaea. Library preparations via Nextera kit and sequencing using an Illumina MiSeq were performed at the Center for Environmental Biotechnology at the University of Tennessee in Knoxville. At total of 22,477,189 reads were produced as a result of 2 Miseq sequencing runs.

Qiime2 was used to trim adaptors and make contigs of bidirectional sequences, denoised using Dada2, and generate Amplicon Sequence Variants (ASVs) at 99% similarity, and classify them with the Silva reference set 132 (Pruesse et al., 2007). Following quality control 16,632,317 (74%) of original reads surviving containing 72,445 unique sequences. Samples with fewer than 20,000 reads were removed from further analysis and ASV’s appearing in fewer than three samples were also removed. All remaining samples were then scaled to even depth of the smallest sequence library size, which was 20,000 reads (McMurdie and Holmes, 2013). Sequences that identified as chloroplast, eukaryote, or failed to classify on the domain level were removed from further analysis. ASV’s for WOR5.16 incubation were agglomerated to the species (97% similarity) level using the command taxa_glom() in the package phyloseq (McMurdie and Holmes, 2013) due to the sequences being produced using two separate Miseq runs.

### Transcriptomics

RNA was extracted from WOR5.16 frozen sediments using the Qiagen RNA Powersoil kit. The concentration of total RNA was determined (Supplementary Table 1) using the Qubit^®^ RNA Assay Kit (Life Technologies). 200–500 ng of total RNA was used to remove the DNA contamination using Baseline-ZERO™ DNase (Epicentre) following the manufacturer’s instructions followed by purification using the RNA Clean & Concentrator columns (Zymo Research). DNA-free RNA samples were used for library preparation using the TruSeq™ RNA LT Sample Preparation Kit (Illumina) according to the manufacturer’s instructions. Following the library preparation, the final concentration of all the libraries (Supplementary Table 1) were measured using the Qubit^®^ dsDNA HS Assay Kit (Life Technologies), and the average library size was determined using the Agilent 2100 Bioanalyzer (Agilent Technologies). The libraries were then pooled in equimolar ratios of 2nM, and 5.5pM of the library pool was clustered using the cBot (Illumina) and sequenced paired end for 500 cycles using the HiSeq 2500 system (Illumina) at Mr. DNA (Shallowater, Texas).

Reads were trimmed using Trimmomatic in paired-end read mode with a minimum quality score of 25 and a maximum of 4 low-quality bases (122). There was an average of 3185420 (minimum = 616402, maximum = 7063586) RNA reads across the ten samples after quality filtering from 0 to 647 days of incubation. Transcriptomic reads were mapped to publicly available assembled metagenomic contigs from the same station in the White Oak River estuary sediments (PRJNA366356) using Bowtie version 2.3.5 with the “sensitive” end-to-end setting (123). Resulting files were converted to bam files using SAMtools version 1.9 and an anvi’o v5.3.0 database was created and each sample profiled against the metagenomic contigs using the anvi’o command anvi-profile and ORF determined by Prodigal (124–126). Gene coverage and detection files were exported using the anvi’o command anvi-export-gene-converage-and-detection, resulting in reads per kilobase per million (RPKM). ORFs were exported as amino acid sequences using anvi’o command anvi-get-aa-sequences-.

#### Gibbs Free Energy Calculations

The Gibbs Free Energy change (△G) associated with hydrogenotrophic methanogenesis, 2H_2_ + CO_2_→ CH_4_ + 2H_2_O, was calculated with the following equation:


△G=△G°+RTln[CH4](aq)[CO2](aq)[H2](aq)4

where, △G° is the standard state Gibbs Free Energy of -194.53 kJ/mol at 18°C, close to the incubation temperature of 21°C (Amend and Shock, 2001), *R* is the gas constant 0.0083145 kJ/molK, *T* is the incubation temperature of 21°C, and aqueous concentrations are used instead of the activities because uncharged constituents have activity coefficients close to 1. The concentration of CO_2_ was estimated at 10 mM, the average sum CO_2_ for the upper 35 cm of station H, White Oak River (Kelley et al., 1990) to provide a high estimate, providing as exergonic an estimate as possible for the △G of hydrogenotrophic methanogenesis in the final three timepoints.

### Data Archiving

16S rRNA gene sequences and RNA transcripts can be found at the NCBI sequence read archive Bioproject PRJNA624356. Geochemistry and qPCR data can be found at www.bco-dmo.org with project number 649807.

## Results

### WOR5.17 Incubation, Methane Added on Day 44, No Resampling

**FIGURE 1:**
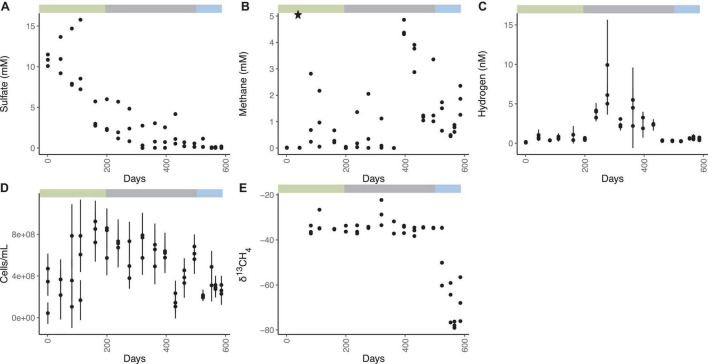
Geochemistry for WOR 5.17 incubation, with **(A)** aqueous sulfate concentrations, **(B)** aqueous methane concentrations, with the star representing the time and concentration of methane added to all bottles, **(C)** aqueous hydrogen concentrations, **(D)** cell abundance, and **(E)** δ^13^CH_4_. Error bars are one standard deviation from triplicate measurements from a single bottle, and are available only for methane, hydrogen, and cell abundance. Green bar shows the time period of sulfate reduction and AOM, gray bar shows the time period of methane cycling through methanogenesis and AOM, and the blue bar shows the time period of methanogenesis.

The WOR5.17 incubation was designed to minimize gas leaks and make subsampling easy by allowing each incubation vial to remain undisturbed until it was destroyed for a timepoint. Sediments from WOR5.17 were incubated in separate butyl-rubber-stoppered glass vials (Figure 1 and Supplementary Table 1) and each remained sealed until it was destroyed for a timepoint. Sulfate decreases from an initial value of 10.8 ± 0.7 mM to 1.0 ± 1.5 mM on day 320, and does not change for the next 3.7 months (1.3 ± 1.4 mM, mean of 12 measurements during days 320–431; Figure 1A). Afterward, sulfate falls to 0.38 ± 0.36 mM for 2 months, averaged across all 9 measurements from days 461–524. Finally, sulfate decreases again to 0.08 ± 0.07 mM for 1 month, averaged across all 9 measurements from days 553–586. Aqueous methane concentrations are initially 0.014 ± 0.011 mM (Figure 1B). After the addition of 5.11 mM methane on day 44, methane decreases gradually to 0.02 ± 0.03 mM by day 200, likely from sulfate-dependent AOM, and then varies between 0.01 and 2.05 mM among replicates and timepoints over days 238 – 320. By day 363, methane drops to < 0.01 mM in all three replicates, increases to 4.51 ± 0.30 mM by day 396, and decreases in a variable way to a low point of 0.47 ± 0.02 mM on day 553, coinciding with the full depletion of sulfate to 0.08 ± 0.07 mM. Methane then increases to a final value of 1.83 ± 0.55 mM on day 586. The high variability of methane when sulfate is low but not absent (days 238–524) is not due to small changes in sulfate concentrations since methane and sulfate do not correlate over this time period (Supplementary Figure 2). Aqueous hydrogen concentrations are < 1.08 nM in the 18 measurements until day 238, when hydrogen increases to a maximum of 7.01 ± 2.58 nM on day 276, even though sulfate is still > 1 mM (Figure 1C). After this, hydrogen declines to an extremely low precise value of 0.30 ± 0.05 nM for 2 months, averaged across all 9 measurements between days 461–524, coinciding with the time that sulfate is stable at ∼0.3 mM. After this, when sulfate decreases to ∼0.07 mM, and methane increases, hydrogen rises slightly to 0.61 ± 0.13 nM for 1 month, averaged across all 9 measurements between days 553–586. The methane added experimentally on day 45 had a δ^13^C-CH_4_ value of -34.9 ‰. The δ^13^C-CH_4_ values are depleted in the lighter isotope slightly at 111 and 323 days, each corresponding to a depletion of aqueous methane concentrations (Figure 1E). This is consistent with the presence of AOM due to kinetic preference for ^12^C over ^13^C, leaving the residual methane ^13^C-enriched (Alperin et al., 1988; Jørgensen and Kasten, 2006; Holler et al., 2009). No large differences in δ^13^C-CH_4_ were observed until days 524–586 when δ^13^C-CH_4_ steadily declines to the lowest value at –79 ‰ on day 568. These values are indicative of the biogenic production of methane, resulting in enrichment of the lighter carbon isotope, ^12^C (Whiticar, 1999). Total cellular abundance in WOR5.17 is 2.06 × 10^8^ ± 1.14 × 10^8^ cells/ml initially, increasing in abundance to 8.32 × 10^8^ ± 2.34 ×10^8^ cells/ml at 161 days, and then declining to 2.68 × 10^8^ ± 8.64 × 10^7^ cells/ml at 586 days followed by a slight increase until the end of the incubation (Figure 1D). At the end of the experiment, autoclaved sediment controls had 5.13 mM sulfate, 0.38 mM methane, 0.62 nM hydrogen, and methane δ^13^C of –34.9. Sulfate and methane concentrations were below detection limit in the sterilized water controls.

**FIGURE 2:**
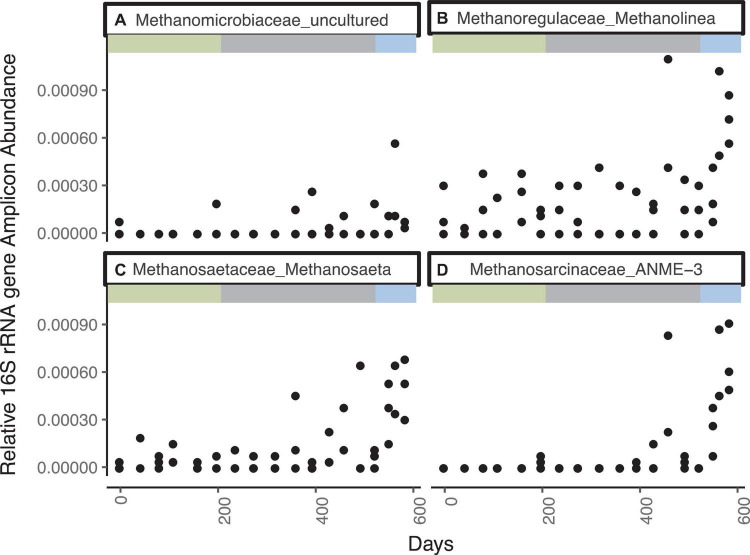
Genera from the Methanomicrobia that increase in 16S rRNA gene amplicon abundance, divided by the total reads in each library, for WOR 5.17 incubation, with **(A)** Methanomicrobiaceae_uncultured, **(B)** Methanoregulaceae_Methanolinea, **(C)** Methanosaetaceae_Methanosaeta, and **(D)** Methanosarcinaceae_ANME-3.

The most abundant genera of the Methanomicrobia are, in order of abundance, *Methanolinea* sp., ANME-3, *Methanosaeta* sp., and uncultured Methanomicrobiaceae, and they all have similar abundance dynamics throughout the incubation (Figure 2 and Supplementary Figure 3). Only two ASVs of ANME-1 are present at five timepoints with low relative abundances (<1× 10^–5^). For the first 363 days, Methanomicrobia are in very low abundance (sums ranging from 0 to 0.08% of total libraries), even though methane was added to the headspace on day 44 and methane concentrations increase and decrease during this time period. The Methanomicrobia increase slightly between days 363 - 431, varying between 0.03 to 0.10% when sulfate drops to ∼1.3 mM. Between days 461–524, Methanomicrobia increase again to a range of 0.02 to 0.32% while sulfate drops again to ∼0.3 mM. The only time interval over which Methanomicrobia are consistently high (0.20 ± 0.90%) is during the final three timepoints, when sulfate is fully depleted, methane is > 0.45 mM, hydrogen is stable at 0.6 nM, and δ^13^C-CH_4_ indicates net methanogenesis.

**FIGURE 3:**
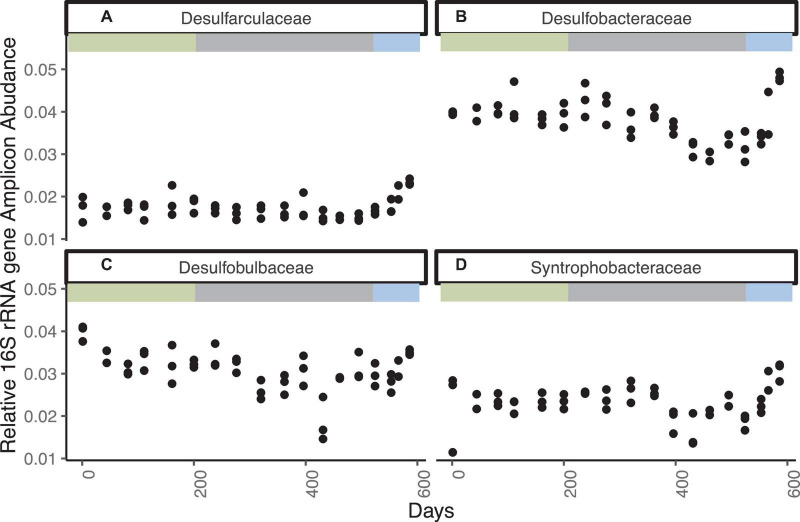
Families with cultured sulfate reducers that increase in relative 16S rRNA gene amplicon abundance for WOR 5.17 incubation, with **(A)** Desulfarculaceae, **(B)** Desulfobacteraceae, **(C)** Desulfobulbaceae, and **(D)** Syntrophobacteraceae.

Bacteria closely related to cultured sulfate reducing bacteria are represented by 1563 ASVs accounting for as much as 11% of the total sequence abundance (Figure 3 and Supplementary Figure 4). The three most abundant ASVs are unclassified members of the family Desulfobulbaceae, which together account for ∼2% of total sequences. Classified and unclassified members of the family Desulfobulbaceae (242 ASVs) account for ∼3.5% of total sequences observed throughout the incubation. The Desulfobacteraceae/Sva0081 sediment group has 121 ASV’s consistently accounting for ∼2–3% of sequence abundance. The genus *Desulfatiglans* in the Desulfarculales represent 1.5% of sequence abundance and 353 ASVs. Desulfobacteraceae/SEEP-SRB1, another putative SRB and partner with anaerobic methane oxidizers (Schreiber et al., 2010), accounts for ∼0.5% of total sequences and 141 unique ASVs. Syntrophobacteraceae, which also contain sulfate reducers, accounts for ∼3.5% of total sequences and 133 ASVs. The relative abundance of these groups does not change noticeably through day 524, with the exception of a decline in all three replicates on day 431, as sulfate decreases from ∼1.3 mM to ∼0.3 mM. Desulfobacteraceae, Desulfobulbaceae, and Syntrophobacteraceae increase in all three replicates in the last three timepoints when sulfate is fully depleted and methanogenesis begins to affect the carbon isotopes. Aerobic sulfur-oxidizing bacteria and aerobic methanotrophs are less than 0.3% of the amplicons and do not increase over time. Aerobic methane and sulfur oxidizers were also present, but in low abundance and with no clear changes with geochemistry (Supplementary Figure 5).

Bacteria comprise the majority of total amplicon sequences with a slight decrease over time, beginning at ∼78% and ending at ∼73% of sequences (Supplementary Figure 6). Proteobacteria are the most abundant bacterial phylum, accounting for approximately 38% of sequences throughout the incubation. Archaeal sequences represent ∼12% of total microbial abundance at the beginning of the incubation and ∼17% of abundance after 586 days (Supplementary Figure 7). Bathyarchaeaota (called Crenarchaeota in the Genome Taxonomy Database taxonomy, and Miscellaneous Crenarchaeotal Group and Marine Benthic Group C, previously) are the most abundant archaeal phylum in the WOR5.17 incubation and gradually increase over time from 5% of sequences to 8% of sequences after 586 days. Lokiarchaeia (also called Deep Sea Archaeal Group and Marine Benthic Group B) increase to 2% of sequences during the final methanogenic portion of the incubation. Thermoprofundales (also called Marine Benthic Group D, Deep Sea Hydrothermal Vent Euryarchaeota Group 1, and Izemarchaea) increase to 2% of sequences during the methane-cycling portion of the incubation and fall off during the methanogenic portion.

### WOR5.16 Incubation, No Amendments, With Resampling

**FIGURE 4:**
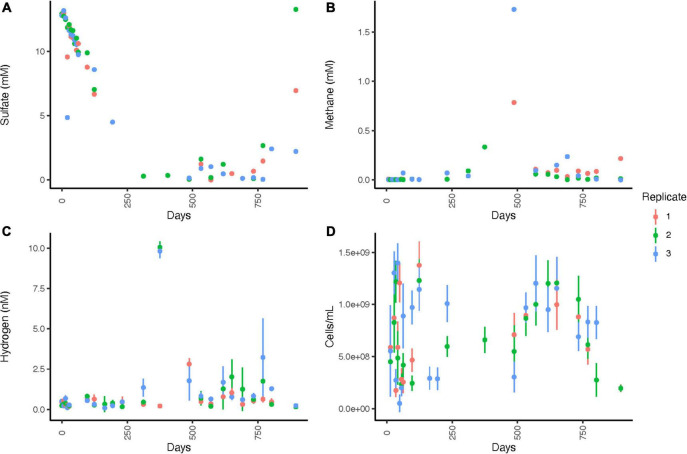
Geochemistry for WOR 5.16 incubation, with **(A)** aqueous sulfate concentrations, **(B)** aqueous methane concentrations, **(C)** aqueous hydrogen concentrations, and **(D)** cell abundance. Error bars are one standard deviation from triplicate measurements from a single bottle, and are available only for methane, hydrogen, and cell abundance. No clear designations for time periods of sulfate reduction, AOM, or methanogenesis were made since it is unclear when oxygen began leaking into the system, δ^13^CH_4_ was not measured for this incubation, and many datapoints were missing due to difficulty pulling sediments through the stopcock, so net processes were less clear than for WOR 5.17 and are therefore not listed. Note that timepoints were weekly for the first 63 days and then moved to longer intervals after that.

WOR5.16 lacks data for some timepoints due to the difficulty of pulling samples through the stopcock for subsampling. Sulfate concentrations decrease from 12.9 ± 0.2 mM to 0.3 mM by day 312 and remain at or below 1.61 mM until day 769, when they gradually increase to a final value of 7.5 ± 5.5 mM by day 895 (Figure 4A and Supplementary Table 1). Methane concentrations are < 0.01 mM in 25 out of the 26 measurements in the first 124 days, show slightly higher variable concentrations in days 124 - 312 where few measurements were made, and peak on day 487 at 1.7 mM (Figure 4B). After this, methane drops to 0.07 ± 0.06 mM, averaged across all 22 datapoints available on days 570–895 except replicate 3 on day 769, which is 8.16 mM. The initial aqueous hydrogen concentration is 0.118 ± 0.401 nM and remains < 1.0 nM until sulfate is depleted at day 312 (Figure 4C). Hydrogen rises to a maximum of 10.1 ± 0.36 nM on day 375, before declining and reaching a very low value of 0.19 ± .03 nM on day 895. Total cellular abundance is 2.64 × 10^8^ ± 1.03 × 10^8^ cells/ml initially, increases to 1.22 × 10^9^± 2.00 × 10^8^ cells/ml by 35 days, before decreasing to a final value of 1.94 × 10^8^ ± 3.29 × 10^7^ by day 895 (Figure 4D).

**FIGURE 5:**
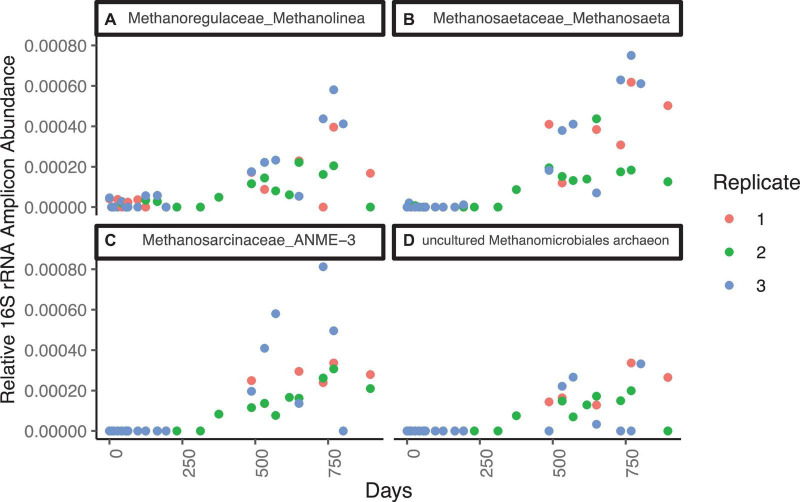
Families from the Methanomicrobia that increase in relative 16S rRNA gene amplicon abundance for WOR 5.16 incubation, with **(A)** Methanoregulaceae_Methanolinea, **(B)** Methanosaetaceae_Methanosaeta, **(C)** Methanosarcinaceae_ANME-3, and **(D)** Methanomicrobiales_uncultured, which was only taxonomically classified at the order level.

The most abundant genera of the Methanomicrobia are, in order of amplicon abundance, *Methanosaeta* sp., ANME-3, and *Methanolinea* sp., and they all have similar abundance dynamics throughout the incubation (Figure 5 and Supplementary Figure 8). Unclassified clades of Methanosarcinales and Methanomicrobiales are well-represented, with 33 ASVs. Five ASVs of ANME-1 are in negligible abundance (3 timepoints at or below 1.0 × 10^–5^). All Methanomicrobia are in very low relative abundance (sums ranging from 0 to 0.01% of total libraries) for the first 312 days, while sulfate is present (Figure 5). After this, Methanomicrobia increase to > 0.05% in all timepoints and replicates. Its relative abundance varies between 0.05 and 0.15% between days 312 and 895, and does not correlate to methane, sulfate, or hydrogen concentrations.

Evidence that oxygen leaked into the three incubation vessels comes from the increase in sulfate concentrations after 500 days. During this time, sulfate reducing bacteria, which are anaerobic, decline in relative abundance (Supplementary Figure 9). Aerobic sulfur oxidizers *Sulfurovum* sp. and *Sulfurimonas* sp. increase in relative abundance when sulfate increased at the end of the incubation (Supplementary Figure 10). Aerobic methanotrophs, *Methylmonaceae* sp. and *Methyloligellaceae* sp. also increase during this time (Supplementary Figure 10). Air contamination was likely during sampling since we could smell sulfide, and if sulfide gas was escaping into the air, then oxygenated air was getting into the bottles. This may have only affected the incubations toward the end of the experiment since during that time free sulfide may have become limited due to pyritization.

Total bacteria decrease from ∼92% of sequences initially to ∼89% after 895 days, with archaea making up the balance (Supplementary Figure 11). Proteobacteria is the most abundant bacterial phylum and accounts for approximately 40% of sequences throughout the timepoints. Bathyarchaeaota (called Crenarchaeota in the GTDB taxonomy) is consistently one of the most abundant archaeal clades in the WOR5.16 incubation and increases over time from 2% of sequences to ∼6% sequences after 895 days (Supplementary Figure 12).

**FIGURE 6:**
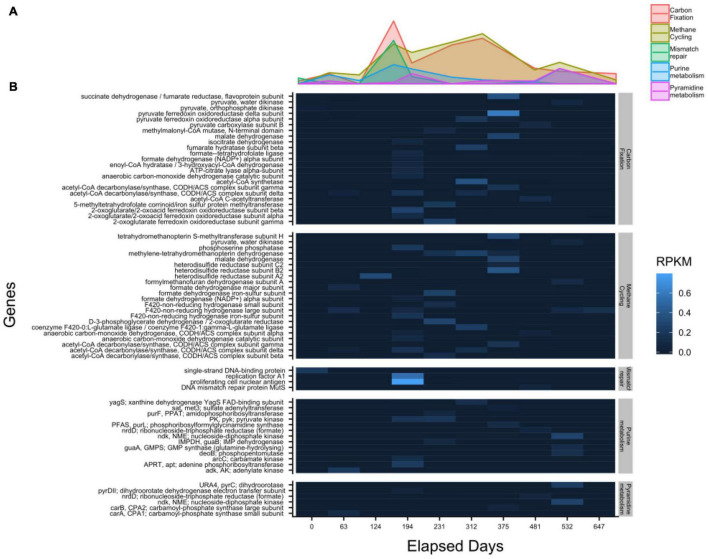
Transcript abundances, shown by function in the WOR 5.16 incubation reveal the highest level of carbon fixation related gene expression occurs prior to sulfate depletion, with **(A)** the summed value of each pathway over time, and **(B)** the individual genes detected in each pathway. Heatmap color corresponding to RPKM (Reads Per Kilobase Mapped).

mRNA transcripts mapping to 41 methane-cycling genes increase in abundance as sulfate approaches depletion at 312 days and peak shortly afterward at 375 days (Figure 6). This agrees with previous studies showing that the highest rate of methane production occurs prior to sulfate depletion (Beulig et al., 2018a). Transcripts for the gene encoding methyl coenzyme M reductase (*mcrA*), a key gene in methane production, were not detected. Transcripts mapped to only three genes in two time points in sulfur-cycling pathways, one of which was exclusive to dissimilatory sulfate reduction, adenylylsulfate reductase subunit A (*aprA*), at 375 days of incubation. Five transcripts mapped to genes related to nitrogen cycling metabolism across 3 samples. None of them were exclusive to energy generation and they included amino acid synthesis genes such as glutamine synthase (*glnA*) and glutamate dehydrogenase (*gdhA*). Transcripts mapped to 42 genes related to carbon fixation. These peak in expression at 194 days but remain actively expressed throughout the incubation. This suggests that microbes may be actively using other carbon pathways, such as the reductive citrate cycle and the reductive acetyl-CoA pathway, however, we cannot accurately distinguish between them since these genes are commonly shared with methanogenesis. Six mRNA transcripts associated with pyrimidine metabolism have the highest expression occurring after 375 days. Conversely, 13 purine metabolism associated genes are more evenly distributed between 63 and 532 days. The expression of genes associated with DNA replication were not detected and only 4 genes associated with DNA mismatch repair were detected while the two highest expression levels are both on day 194; proliferating cell nuclear antigen and replication factor A1, respectively. The expression of only one gene, *motB*, associated with flagellar motility occurs at 124 days. No other genes recruited mRNA reads in more than two timepoints.

## Discussion

In both sets of triplicate WOR sediment incubations, methane production begins well before sulfate is depleted and oscillates between production and consumption, with variable increases in methanogenic groups. The highest concentrations of hydrogen and methane are reached during this period of methane cycling. It is only after sulfate is fully depleted that methane increases reliably, isotopes show net methanogenesis, and methanogenic groups increase consistently. This is the only time that sulfate reducing bacteria increase in abundance. A similar increase and decrease in methane right after sulfate depletion was also observed for CLB sediments, but it lasted only 18 days, vs 315 in our WOR incubations (Kevorkian et al., 2018). Reaction transport models suggest that lower organic matter fluxes in the WOR support slower microbial metabolic rates (Martens et al., 1998). Despite starting with 1–8 mM less sulfate than CLB, WOR sediments take much longer for sulfate to decrease to ∼1 mM and begin producing methane: 312–323 days in WOR sediments vs. 40 days (Alperin et al., 1992) and 68 days (Kevorkian et al., 2018) for CLB sediments, with all incubations at 21.5°C.

In our WOR incubations, once sulfate concentrations decrease to less than ∼5 mM after ∼200 days, all replicates in both incubations show a consistent pattern of a peak in hydrogen, followed by a peak in methane about 4 months later, and then consumption of that methane, all occurring in the 315 days before sulfate is fully depleted. Hydrogen increases at a rate of 0.09 nM H_2_/day (WOR5.17, days 200-276, *n* = 9, *R*^2^= 0.82) suggesting that sulfate reducers lose thermodynamic control of hydrogen even though sulfate is still present (1–5 mM), possibly because, at these low concentrations of sulfate, diffusion limits the concentration of sulfate at the cell surfaces (Hoehler et al., 2001). The subsequent decrease in hydrogen is likely driven by hydrogenotrophic methanogens converting hydrogen to methane at a rate of 0.13 mM CH_4_/day (WOR5.17, days 362–396, *n* = 6, *R*^2^= 0.99) even though sulfate concentrations are still 0–3 mM. At this time, methane-cycling archaea (designated here as archaea belonging to genera whose cultured members are methanogens, or that have been shown in previous studies to have full methanogenic genetic pathways) increase sporadically, and RNA expression of methane-cycling genes in the WOR5.16 incubation are present, suggests that methane-cycling occurs well before sulfate is depleted. However, during this time δ^13^C-CH_4_ shows no net AOM or methanogenesis. The subsequent decrease in methane at a rate of 0.05 mM CH_4_/day (WOR5.17, days 396–461, *n* = 9, *R*^2^ = 0.89) is not accompanied by any major changes in methane-cycling population or δ^13^C-CH_4_.

This 11.5-month period of overlap between methanogenesis, AOM, and sulfate reduction is likely not an artifact of sampling methods since it was observed in incubation 5.16, which was subsampled from the same bottles over time, and incubation 5.17 where bottles were incubated separately and destroyed for each timepoint. Diffusion limitation on the microscale likely creates heterogeneity in space and time in the hydrogen concentrations that cells experience, meaning that some cells oxidize methane while others produce it, or individual cells oscillate between production and oxidation of methane. It is also possible that cryptic sulfur cycling extended the length of this transition period and allows methane to cycle. In cryptic sulfur cycling, sulfide produced by sulfate reducing bacteria, is reoxidized by oxidized compounds such as ferric iron or barite dissolution (Borowski et al., 1996; Knab et al., 2008; Holmkvist et al., 2011; Xiao et al., 2017; Pellerin et al., 2018; Beulig et al., 2019). Radiotracer experiments in marine sediment columns have shown that when sulfate concentrations are low, the rate of reoxidation roughly balances the rate of depletion (Holmkvist et al., 2011; Pellerin et al., 2018; Jørgensen et al., 2019), which would explain why sulfate did not change much over this interval in WOR5.17. The cessation of sulfate reduction and commencement of steady methanogenesis around day 553, therefore, could have occurred because sulfide became fully mineral-bound so it was no longer available for reoxidation.

For the latter part of the incubations, we consider only the results from WOR5.17, since WOR5.16 shows evidence of oxygen contamination during these late stages. In the final 3 months of this methane- and sulfur-cycling period (days 461–553), the mean sulfate concentration drops to 0.08 ± 07 mM (*n* = 9) and hydrogen drops to the lowest and most stable value of the entire experiment (0.30 ± 0.05 nM, *n* = 9). Even though the sediment in all nine bottles sampled during this time period have been separated for a year and a half, they all reach this precise low hydrogen concentration during this same time interval. Methane concentrations and the relative abundance of 16S rRNA genes of methane-cycling archaea, on the other hand, are variable between individual bottles during this interval, with no trends.

It is not until sulfate is depleted to ∼0.07 mM (day 553) that δ^13^C-CH_4_ finally decreases to -80‰, showing evidence of net methanogenesis, and methane-cycling populations consistently increase in relative abundance in all bottles. During this month, methanogenesis rates are 0.04 mM/day (WOR5.17, *n* = 9, *R*^2^= 0.79). This rate is a third of the methanogenesis rate observed after the hydrogen peak during the time of methane- and sulfur-cycling, reflecting the slower methanogenesis rates at lower hydrogen concentrations. This suggests that the period of methane-cycling has stopped, and methane is mainly produced, not oxidized. These rates of methane production (both during methane-cycling and methane production) are much higher than rates inferred from reaction transport modeling at this site of 0.007 mM/day (Lloyd et al., 2011) and 0.002 mM/day (Martens et al., 1998). However, they are quite similar to *in situ* radiotracer rate measurements made in Aarhus Bay, Denmark (0.06 mM/day), suggesting that rates from reaction transport models may underestimate the real metabolic rates.

Throughout the methane-cycling and methanogenic phases of the incubations, the only methane-cycling archaea that increase belong to families whose only cultured relatives are hydrogenotrophic and aceotclastic methanogens, *Methanoregulaceae* (*Methanolinea* sp. and *Methanoregula* sp.), *Methanosarcinaceae* (*Methanosaeta* sp., ANME-3, and other uncultured genera), and *Methanomicrobiaceae* (uncultured genera). ANME-3 has been implicated as a methanotroph based on its presence in methane seeps performing AOM (Knittel et al., 2005; Parkes et al., 2007), and it is in the genus *Methanococcoides*, whose cultured relatives are all methanogens. These different clades of methane-cycling archaea have the same patterns of growth during these incubations, suggesting that they all benefit intermittently during the period of methane-cycling and then consistently during the period of methanogenesis. It is possible that each of these archaea is capable of reversing between methanogenesis and AOM, taking advantage of hydrogenotrophic methanogenesis or AOM, depending on which one is exergonic. Even members of the *Methanosarcinales*, whose cultured members are acetoclastic methanogens that do not use hydrogen directly, still require adequate hydrogen concentrations to respire since they “leak” hydrogen as a metabolic intermediate when they are in very low hydrogen concentrations (Finke et al., 2007).

**FIGURE 7:**
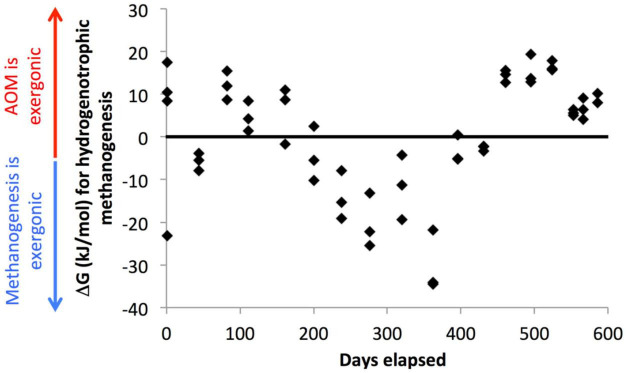
Gibbs free energy associated with hydrogenotrophic methanogenesis in WOR 5.17 incubation, with positive values meaning that AOM through reverse methanogenesis is exergonic and negative values meaning that hydrogenotrophic methanogenesis is exergonic.

These populations of methane-cycling archaea are very different than those found *in situ* at this site, which are almost entirely ANME-1 (Lloyd et al., 2011; Lazar et al., 2015; Kevorkian et al., 2021). The methane-cycling archaea growing in the WOR incubations are more similar to those that grow in CLB incubations, *Methanosarcinaceae* (*Methanosaeta* sp. and uncultured genera) and *Methanomicrobiaceae* (uncultured genera) (Kevorkian et al., 2018). ANME-1 are in very low abundance in WOR5.16 and WOR5.17, and do not increase in relative abundance during the incubations, even in WOR5.17 where the early addition of methane enabled AOM. The methane-cycling archaea in the WOR incubations may simply have grown faster than ANME-1, which has a doubling time of 7 months (Nauhaus et al., 2007). In fact, the addition of methane early in the incubations of WOR5.17 makes very little effect on the progress of sulfate reduction, or the microbial community at all, even though it is consumed via AOM. Here, sulfate concentrations are consistently high (>4 mM), which keeps hydrogen consistently low (<1 nM), making AOM using reverse methanogenesis exergonic (Figure 7). The fact that methane-cyclers do not grow when sulfate is > 5 mM and methane only decreases, grow intermittently when sulfate is 0–5 mM and methane and hydrogen are variable, and grow consistently when sulfate is ∼0 mM and methane only increases, suggests that methanogenesis may provide more energy for growth than AOM for these populations. This implies that they only grow during periods of methanogenesis, and simply meet maintenance energy needs during periods of AOM.

Curiously, after the final depletion in sulfate, when net methanogenesis becomes consistent, the small hydrogen concentration increase to 0.61 nM ± 0.13 (*n* = 9) is not sufficient to make hydrogenotrophic methanogenesis exergonic (Figure 7). It is possible, therefore, that other types of methanogenesis, such as acetoclastic or methylotrophic support methane production here, but this would not explain which terminal electron accepting process keeps hydrogen concentrations so low. Desulfarculaceae, Desulfobulbaceae, and Syntrophobacteraceae increase along with the methane-cycling archaea during this time, suggesting that a syntrophic relationship where these bacteria ferment organic matter and donate reduced compounds to methanogens may allow them to push close to their thermodynamic limits as has been predicted previously (Jackson and McInerney, 2002; D’Hondt et al., 2004; Wang et al., 2008, 2010) or to supplement their metabolism with direct electron transfer. This is the only time during the incubation that these sulfate reducing bacteria increase in relative abundance, even though sulfate reduction has been occurring through much of the incubation. The only other major clades that increase in relative abundance during the incubation are Bathyarchaeota, which increase steadily irrespective of geochemical shifts, and Lokiarchaeota and Thermoprofundales, which have not been found to contain methanogenic genes. Unlike the methanogens, the sulfate-reducing bacteria in the WOR5.16 and WOR5.17 incubations are from the same genera found *in situ* (Kevorkian et al., 2021): SEEP-SRB1, *Desulfatiglans* sp., and *Desulfobulbus* sp. This suggests that if the sulfate reducers form syntrophic relationships with methanogens, these relationships are not species specific. Syntrophy with the newly grown methanogens may open a new niche that the sulfate reducers are able to grow into, allowing them to finally be able to remove the last bit of sulfate. This offers an alternative explanation to the waning sulfide availability due to mineralization discussed above.

Our results agree with those inferred from down-core studies (Beulig et al., 2018b; Kevorkian et al., 2021) that the transition between sulfate reduction/AOM to methanogenesis in marine sediments goes through a substantial transitional period of methane-cycling. Here, sulfate concentrations are low and sediments shift between AOM and methanogenesis. This methane-cycling supports intermittent growth of methanogens including *Methanoregulaceae* (*Methanolinea* sp. and *Methanoregula* sp.), *Methanosarcinaceae* (*Methanosaeta* sp., ANME-3, and other uncultured genera), and *Methanomicrobiaceae* (uncultured genera), all of which begin to increase consistently in relative abundance when sulfate is finally depleted and methanogenesis becomes continuous. Since these are the only methane-cycling archaea present, and they all have the same relative abundance patterns, it is likely that they reverse between AOM and methanogenesis, depending on which process is exergonic. Reversals between AOM and methanogenesis have been observed for these clades previously although only methanogenesis has been shown to support growth for them (Valentine et al., 2000). Our results support that they grow only from methanogenesis, but they also perform AOM. Growth of these organisms under conditions of continuous methanogenesis in turn benefits sulfate reducing communities (Desulfarculaceae, Desulfobulbaceae, and Syntrophobacteraceae) that only grow after sulfate depletion, possibly due to syntrophy with methanogens.

## Data Availability Statement

16S rRNA gene sequences and RNA transcripts can be found at the NCBI sequence read archive Bioproject PRJNA624356. Geochemistry and qPCR data can be found at www.bco-dmo.org with project number 649807.

## Author Contributions

RK and KL conceived of the experiments, analyzed data, and wrote the manuscript. RK obtained samples and conducted the experiments. RW and RP made substantial data contributions. KS performed data analysis and visualization. KL advised other authors and obtained funding. All authors contributed to the article and approved the submitted version.

## Conflict of Interest

The authors declare that the research was conducted in the absence of any commercial or financial relationships that could be construed as a potential conflict of interest.

## Publisher’s Note

All claims expressed in this article are solely those of the authors and do not necessarily represent those of their affiliated organizations, or those of the publisher, the editors and the reviewers. Any product that may be evaluated in this article, or claim that may be made by its manufacturer, is not guaranteed or endorsed by the publisher.
