# Supplementary material for: Cryptic Methane-Cycling by Methanogens During Multi-Year Incubation of Estuarine Sediment
Source: Front Microbiol. 2022 Mar 17;13:847563. doi: 10.3389/fmicb.2022.847563 (PMC8969600; doi:10.3389/fmicb.2022.847563)
Supplement: Supplementary file 1 [file Table_1.DOCX]

**Figure S1.** Pictures of incubation set ups. A) WOR 5.16 experimental setup, with unamended sediments placed into three 2L stoppered bottles that were subsampled repeatedly over time. Only two of the three replicates are shown in the picture. B) WOR 5.17 experimental set-up with individual bottles destructively sampled; the two bottles on their own are autoclave-killed controls, and clear bottles represent negative controls of distilled water.


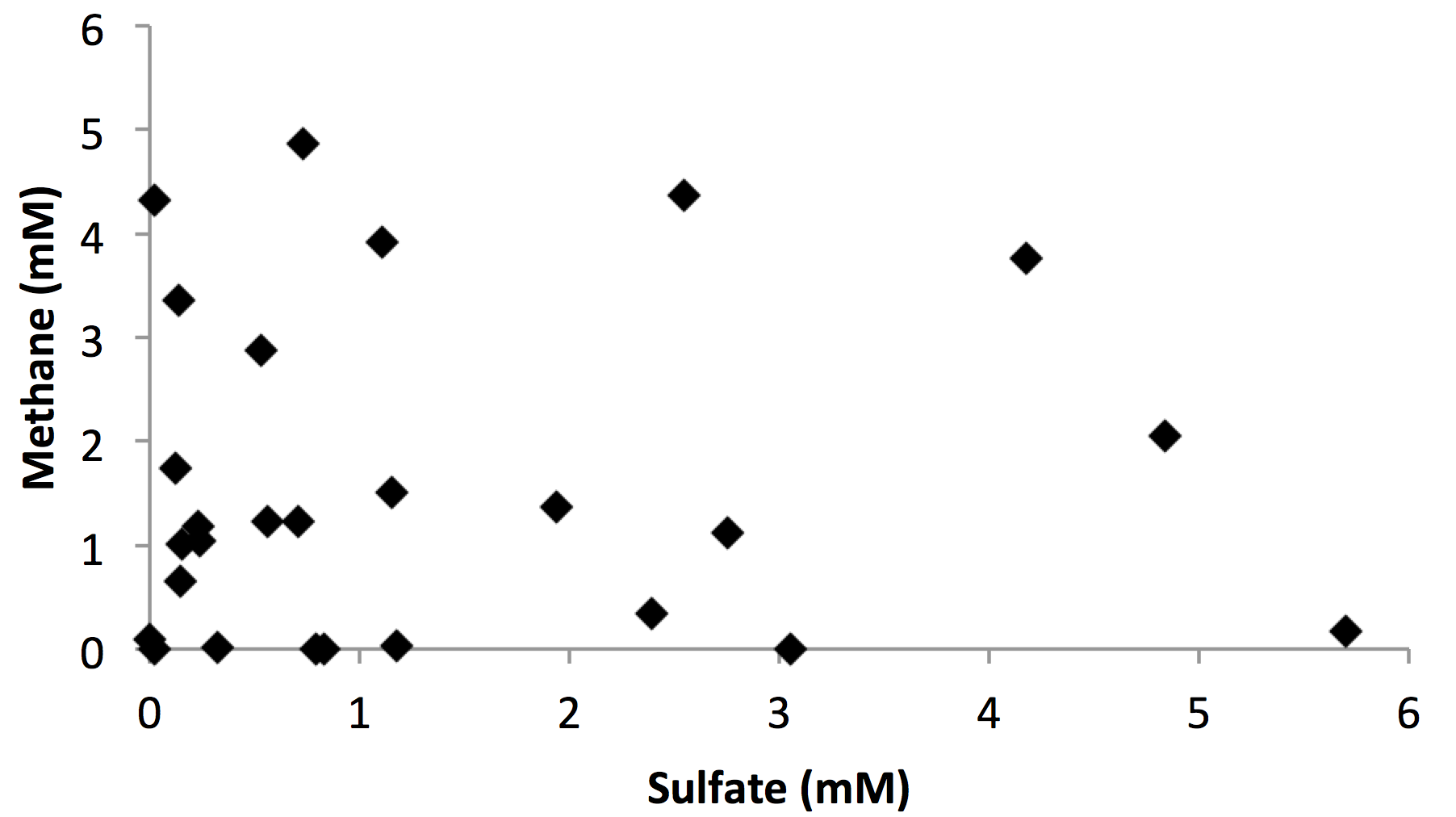


**Figure S2.** Sulfate and methane concentrations do not correlate through the period of methane-cycling days 238-524 in WOR 5.17. Due to sampling difficulties, only 3 measurements were available that had both methane and sulfate in the WOR 5.16 incubation.

**Figure S3.** Methanomicrobia by genus, 16S rRNA relative abundance over the 586-day incubation from WOR 5.17.

**Figure S4.** Families with cultured sulfate reducers by genus, 16S rRNA relative abundance over the 586-day incubation from WOR 5.17.

**Figure S5.** Genera with cultured aerobic methanotroph/methylotrophs and aerobic sulfur oxidizers, 16S rRNA relative abundance over the 586-day incubation from WOR 5.17.

**Figure S6.** Bacteria by phylum, 16S rRNA relative abundance over the 586-day incubation from WOR5.17.

A

B

**Figure S7**. Archaea by A) phylum and B) family, 16S rRNA relative abundance over the 586-day incubation from WOR 5.17.

Fractional Read Abundance

**Figure S8.** Methanomicrobia by genus, 16S rRNA relative abundance over the 895-day incubation from WOR 5.16.

A


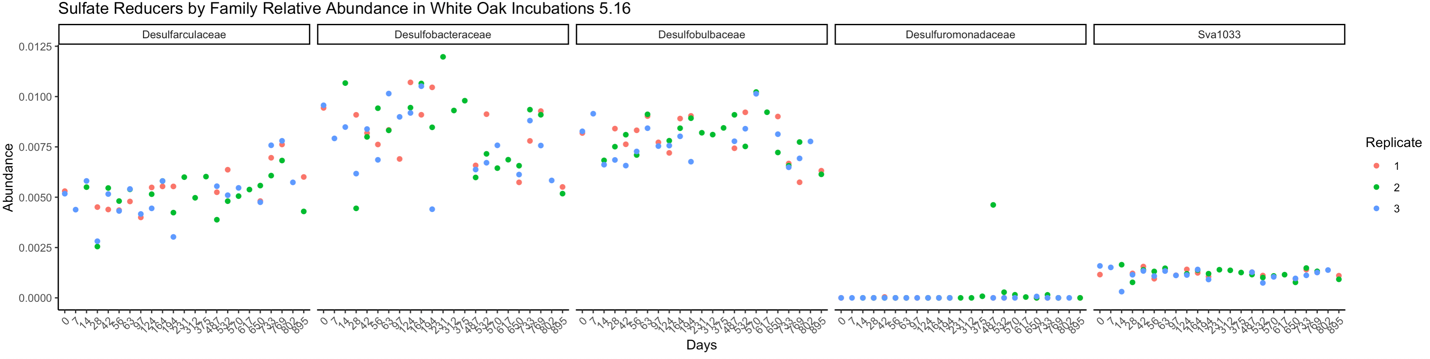


B

**Figure S9.** Families with cultured sulfate reducers by A) family and B) genus, 16S rRNA relative abundance, over the 895-day incubation from WOR 5.16.

**Figure S10.** Genera with cultured aerobic methanotroph/methylotrophs and aerobic sulfur oxidizers, 16S rRNA relative abundance over the 895-day incubation from WOR 5.16.

**Figure S11.** Bacteria by phylum, 16S rRNA relative abundance over the 895-day incubation from WOR 5.16.


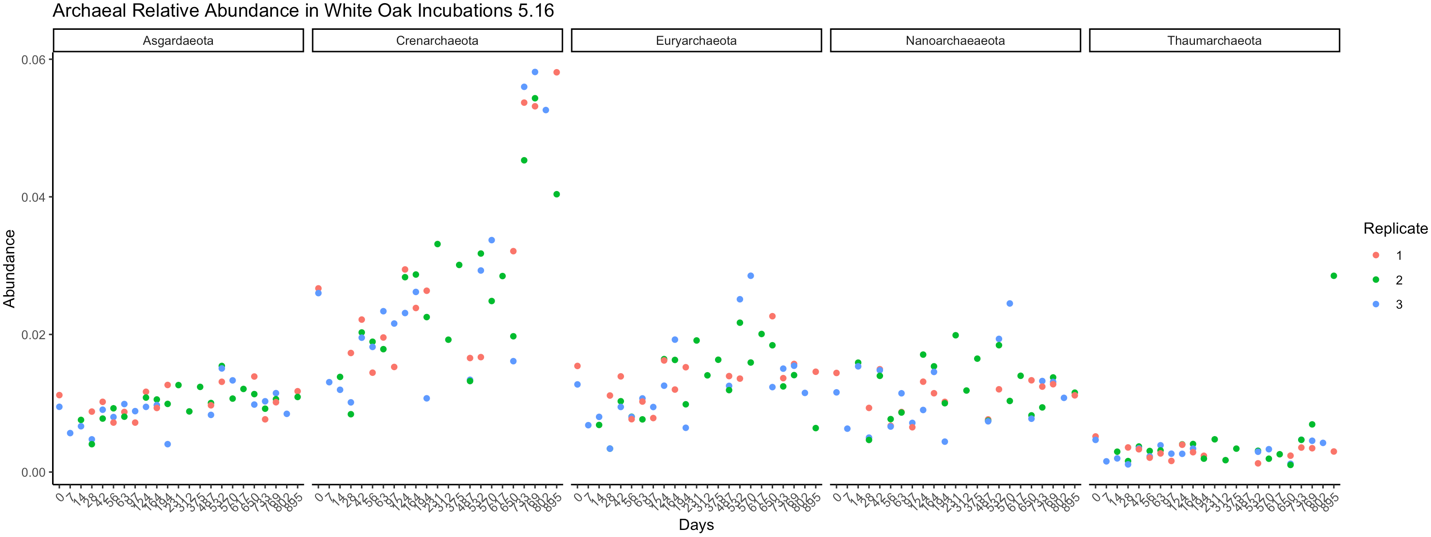


A

B

**Figure S12.** Archaea 16S rRNA phyla relative abundance at the A) phylum level and B) family level over the 895-day incubation from WOR 5.16.

Table S1.4 Data used for incubation analysis. Replicate 0 is the autoclaved control.

| ID | MiseqRun | Experiment | Date | Elapsed time | Replicate | H2 nM | H2 std | CH4 mM | CH4 STD | Isotope ch4 | Iso std | so4 mM | Cells ml | Std dev cells |
| --- | --- | --- | --- | --- | --- | --- | --- | --- | --- | --- | --- | --- | --- | --- |
| 0_0 | 2 | WOR5.17 | 5/26/2017 | 1 | 0 | 3.6807714 | 0.66089945 | 0.00457544 |  |  |  | 9.42060581 | 228666667 | 104364231 |
| 0_1 | 2 | WOR5.17 | 5/26/2017 | 1 | 1 | 0.17274549 | 0.01351508 | 0.01970958 |  |  |  | 11.5006797 | 346266667 | 135803622 |
| 0_2 | 2 | WOR5.17 | 5/26/2017 | 1 | 2 | 0.16063981 | 0.04205351 | 0.00668718 |  |  |  | 10.0874606 | 43555555.6 | 102346767 |
| 0_3 | 2 | WOR5.17 | 5/26/2017 | 1 | 3 | 0.07347361 | 0.01503713 | 0.01126261 |  |  |  | 10.8569084 | 470400000 | 143702609 |
| 1_1 | 2 | WOR5.17 | 7/8/2017 | 44 | 1 | 1.05077966 | 0.68181677 | 0.01513414 |  |  |  | 9.18977147 | 366955556 | 191956308 |
| 1_2 | 2 | WOR5.17 | 7/8/2017 | 44 | 2 | 0.74053341 | 0.00324932 | 0.01020674 |  |  |  | 13.6653928 |  |  |
| 1_3 | 2 | WOR5.17 | 7/8/2017 | 44 | 3 | 0.56231261 | 0.26844975 | 0.00668718 |  |  |  | 10.9492421 | 216688889 | 234071377 |
| 3_1 | 2 | WOR5.17 | 8/15/2017 | 82 | 1 | 0.38521972 | 0.07758887 | 0.23862664 |  | -37.1366 | 0.29003845 | 7.90992331 | 356066667 | 205235112 |
| 3_2 | 2 | WOR5.17 | 8/15/2017 | 82 | 2 | 0.35783024 | 0.11506268 | 0.68279599 |  | -33.6164 | 0.07450705 | 7.7627023 | 104533333 | 203443339 |
| 3_3 | 2 | WOR5.17 | 8/15/2017 | 82 | 3 | 0.3552012 | 0.07511895 | 2.81565359 |  | -36.457 | 0.0528583 | 14.693888 | 785088889 | 303239697 |
| 4_1 | 2 | WOR5.17 | 9/13/2017 | 111 | 1 | 0.55327516 | 0.32739973 | 0.05167898 | 0.00010354 | -34.772 | 0.16752512 | 7.2276796 | 605422222 | 164508112 |
| 4_2 | 2 | WOR5.17 | 9/13/2017 | 111 | 2 | 0.85574289 | 0.41810063 | 0.96553454 | 0.00117904 | -26.607 | 0.18222514 | 15.7813742 | 785088889 | 347037665 |
| 4_3 | 2 | WOR5.17 | 9/13/2017 | 111 | 3 | 0.68407488 | 0.27168433 | 2.17066947 | 0.00272772 | -34.994 | 0.04068784 | 8.5306112 | 167688889 | 191582139 |
| 5_1 | 2 | WOR5.17 | 11/2/2017 | 161 | 1 | 1.0796358 | 1.10106095 | 0.21445895 | 0.00038777 | -35.26 | 0.14454296 | 2.98289261 | 721933333 | 184661094 |
| 5_2 | 2 | WOR5.17 | 11/2/2017 | 161 | 2 | 0.39512993 | 0.04328312 | 0.27264912 | 0.002856 | -35.367 | 0.04669582 | 2.74769807 | 850422222 | 163554716 |
| 5_3 | 2 | WOR5.17 | 11/2/2017 | 161 | 3 | 0.39301462 | 0.03611717 | 0.66871773 | 0.00235992 | -35.105 | 0.03285575 | 5.72725641 | 923377778 | 198783807 |

Table S1 Continued.

| ID | MiseqRun | Experiment | Date | Elapsed time | Replicate | H2 nM | H2 std | CH4 mM | CH4 STD | Isotope ch4 | Iso std | so4 mM | Cells ml | Std dev cells |
| --- | --- | --- | --- | --- | --- | --- | --- | --- | --- | --- | --- | --- | --- | --- |
| 6_1 | 2 | WOR5.17 | 12/11/2017 | 200 | 1 | 0.48233233 | 0.11465513 | 0.04615326 | 0.00106844 | -36.3248 | 0.31594256 | 2.35194542 | 838444444 | 194555340 |
| 6_2 | 2 | WOR5.17 | 12/11/2017 | 200 | 2 | 0.67263811 | 0.16448056 | 0 | 0 | -36.3468 | 0.16432955 | 2.20831517 | 572755556 | 165400407 |
| 6_3 | 2 | WOR5.17 | 12/11/2017 | 200 | 3 | 0.41240318 | 0.07749539 | 0 | 0 | -33.677 | 0.0869885 | 6.00169279 | 859133333 | 188174627 |
| 7_1 | 2 | WOR5.17 | 1/18/2018 | 238 | 1 | 4.1578716 | 0.72741865 | 0.1769169 | 0.00188306 | -36.3614 | 0.33146991 | 5.69904332 | 671844444 | 147763577 |
| 7_2 | 2 | WOR5.17 | 1/18/2018 | 238 | 2 | 3.99732712 | 1.09969242 | 0.03261465 | 6.6561E-05 | -37.156 | 0.16557023 | 1.17725512 | 712133333 | 230254259 |
| 7_3 | 2 | WOR5.17 | 1/18/2018 | 238 | 3 | 3.24313292 | 0.47266769 | 1.36218974 | 0.0045183 | -33.5484 | 0.07614985 | 1.93387879 | 731733333 | 149722052 |
| 8_1 | 2 | WOR5.17 | 2/26/2018 | 276 | 1 | 6.10515359 | 1.32259303 | 2.05331538 | 0.00130677 | -34.186 | 0.26777976 | 4.83726179 | 732822222 | 186905814 |
| 8_2 | 2 | WOR5.17 | 2/26/2018 | 276 | 2 | 9.9189324 | 5.73955423 | 0.34433097 | 0.00050075 | -34.7356 | 0.05096371 | 2.39041781 | 495444444 | 212412747 |
| 8_3 | 2 | WOR5.17 | 2/26/2018 | 276 | 3 | 5.01216577 | 1.38027457 | 0.00608885 | 7.5978E-05 | -34.4482 | 0.05273708 | 0.83356844 | 378933333 | 101359283 |
| 9_1 | 2 | WOR5.17 | 4/11/2018 | 320 | 1 | 2.30660956 | 0.15040234 | 0.08763722 | 0.00067065 | -28.7504 | 0.26930893 | 0 | 768755556 | 235833290 |
| 9_2 | 2 | WOR5.17 | 4/11/2018 | 320 | 2 | 3.07250756 | 0.1917627 | 0.01010116 | 0.0002042 | -22.2618 | 0.13936355 | 0.32316807 | 573844444 | 163599706 |
| 9_3 | 2 | WOR5.17 | 4/11/2018 | 320 | 3 | 2.10541889 | 0.50886701 | 1.11746252 | 0.00392049 | -33.4748 | 0.01756986 | 2.75205827 | 790533333 | 210204090 |
| 10_1 | 2 | WOR5.17 | 5/23/2018 | 362 | 1 | 5.49492395 | 1.49471916 | 0.00022056 | 1.4092E-06 |  |  | 0.02436585 | 492177778 | 171773792 |
| 10_2 | 2 | WOR5.17 | 5/23/2018 | 362 | 2 | 4.49545187 | 5.09939613 | 0.00011849 | 1.4454E-06 | -31.796 | 0.24391187 | 3.05111698 | 654422222 | 190377925 |
| 10_3 | 2 | WOR5.17 | 5/23/2018 | 362 | 3 | 2.19379115 | 0.1684832 | 0 | 0 | -37.1868 | 0.10622241 | 0.7899664 | 700155556 | 202042800 |
| 11_1 | 2 | WOR5.17 | 6/26/2018 | 396 | 1 | 3.2575035 | 0.6544481 | 4.31440252 | 0.00567562 | -36.9868 | 0.71746861 | 0.02487881 | 620666667 | 193353784 |
| 11_2 | 2 | WOR5.17 | 6/26/2018 | 396 | 2 | 3.25514101 | 0.73885805 | 4.37095023 | 0.00259726 | -33.6876 | 0.15242802 | 2.54430737 | 638088889 | 150000330 |

Table S1 Continued.

| ID | MiseqRun | Experiment | Date | Elapsed time | Replicate | H2 nM | H2 std | CH4 mM | CH4 STD | Isotope ch4 | Iso std | so4 mM | Cells ml | Std dev cells |
| --- | --- | --- | --- | --- | --- | --- | --- | --- | --- | --- | --- | --- | --- | --- |
| 11_3 | 2 | WOR5.17 | 6/26/2018 | 396 | 3 | 1.89351689 | 1.17946562 | 4.86028736 | 0.00198612 | -34.2054 | 0.11570653 | 0.7309754 | 576022222 | 133034203 |
| 12_1 | 2 | WOR5.17 | 7/31/2018 | 431 | 1 | 2.32829004 | 0.71682253 | 3.76687522 | 0.00274569 | -35.7984 | 0.04212185 | 4.17297186 | 234111111 | 120433073 |
| 12_2 | 2 | WOR5.17 | 7/31/2018 | 431 | 2 | 2.34096536 | 0.20775725 | 3.91035624 | 0.01025481 | -38.2966 | 0.14153904 | 1.11056965 | 106711111 | 114110511 |
| 12_3 | 2 | WOR5.17 | 7/31/2018 | 431 | 3 | 2.45192577 | 0.21117407 | 2.87619014 | 0.0058765 | -34.534 | 0.21122263 | 0.5334838 | 143733333 | 95374012.7 |
| 13_1 | 2 | WOR5.17 | 8/30/2018 | 461 | 1 | 0.38284348 | 0.0612127 | 1.23230599 | 0.00487245 | -34.562 | 0.22304017 | 0.7104568 | 332111111 | 140722719 |
| 13_2 | 2 | WOR5.17 | 8/30/2018 | 461 | 2 | 0.30344062 | 0.00484543 | 1.0441382 | 0.00054924 | -34.562 | 0.14350087 | 0.23775937 | 454066667 | 115207007 |
| 13_3 | 2 | WOR5.17 | 8/30/2018 | 461 | 3 | 0.28387145 | 0.03157657 | 1.18111975 | 0.00965441 | -34.3226 | 0.08299277 | 0.22826951 | 385466667 | 102729552 |
| 14_1 | 2 | WOR5.17 | 10/3/2018 | 495 | 1 | 0.35848056 | 0.02882636 | 1.01128891 | 0.00453992 | -34.6214 | 0.17361538 | 0.15132473 | 560777778 | 140722719 |
| 14_2 | 2 | WOR5.17 | 10/3/2018 | 495 | 2 | 0.25070938 | 0.03349131 | 3.3576669 | 0.01224944 | -34.807 | 0.13986958 | 0.13593578 | 682733333 | 115207007 |
| 14_3 | 2 | WOR5.17 | 10/3/2018 | 495 | 3 | 0.34554707 | 0.02428001 | 1.23184844 | 0.00184995 | -34.567 | 0.10860018 | 0.56426171 | 614133333 | 102729552 |
| 15_1 | 2 | WOR5.17 | 11/1/2018 | 524 | 1 | 0.28929202 | 0.00872111 | 1.50578807 | 0.00394785 | -34.6436 | 0.98782655 | 1.15160686 | 215600000 | 52603512.3 |
| 15_2 | 2 | WOR5.17 | 11/1/2018 | 524 | 2 | 0.24603121 | 0.04062653 | 1.73303478 | 0.00247925 | -60.2842 | 0.07308351 | 0.12311165 | 2.03E+08 | 3.68E+07 |
| 15_3 | 2 | WOR5.17 | 11/1/2018 | 524 | 3 | 0.23978081 | 0.03726528 | 0.6561646 | 0.00473321 | -50.1256 | 0.01929508 | 0.14619508 | 1.93E+08 | 3.36E+07 |
| 16_1 | 2 | WOR5.17 | 11/30/2018 | 553 | 1 | 0.65057029 | 0.03080142 | 0.44545986 | 0.00137448 | -64.3445 | 0.65070907 | 0.08720408 |  |  |
| 16_2 | 2 | WOR5.17 | 11/30/2018 | 553 | 2 | 0.63389689 | 0.03348511 | 0.48992372 | 0.00290853 | -59.123 | 0.13227812 | 0.00512965 | 310333333 | 154832823 |
| 16_3 | 2 | WOR5.17 | 11/30/2018 | 553 | 3 | 0.57828904 | 0.00734584 | 0.47267785 | 0.00049855 | -76.6734 | 0.0502225 | 0.08720408 | 487822222 | 152723117 |
| 17_1 | 2 | WOR5.17 | 12/13/2018 | 566 | 1 | 0.84722307 | 0.30517003 | 0.8740258 | 0.00042734 | -78.0078 | 0.22746795 | 0.01538896 | 290733333 | 89296672.9 |

Table S1 Continued.

| ID | MiseqRun | Experiment | Date | Elapsed time | Replicate | H2 nM | H2 std | CH4 mM | CH4 STD | Isotope ch4 | Iso std | so4 mM | Cells ml | Std dev cells |
| --- | --- | --- | --- | --- | --- | --- | --- | --- | --- | --- | --- | --- | --- | --- |
| 17_2 | 2 | WOR5.17 | 12/13/2018 | 566 | 2 | 0.49471758 | 0.04639731 | 0.78134387 | 0.00047283 | -79.068 | 0.0543829 | 0.01795378 | 313600000 | 88156183.2 |
| 17_3 | 2 | WOR5.17 | 12/13/2018 | 566 | 3 | 0.60938422 | 0.03991032 | 0.61005828 | 0.00085469 | -76.2786 | 0.08759737 | 0.1282413 | 274400000 | 80292110.4 |
| 18_1 | 2 | WOR5.17 | 1/2/2019 | 586 | 1 | 0.73125827 | 0.02058767 | 2.35693669 | 0.00155982 | -56.5672 | 0.12423734 | 0.1974916 | 313600000 | 88156183.24 |
| 18_2 | 2 | WOR5.17 | 1/2/2019 | 586 | 2 | 0.54798397 | 0.06977394 | 1.86419731 | 0.00250509 | -68.0174 | 0.05608297 | 0.16158404 | 260244444 | 66717333.2 |
| 18_3 | 2 | WOR5.17 | 1/2/2019 | 586 | 3 | 0.38153875 | 0.07220885 | 1.26117817 | 0.00123849 | -76.079 | 0.02056696 | 0.00512965 | 228666667 | 104364231 |
| 18_0 | 2 | WOR5.17 | 1/2/2019 | 586 | 0 | 0.62433886 | 0.0624173 | 0.38245961 | 0.00211475 | -34.91 | 0.03338413 | 5.13221678 | 699066667 | 143702609 |
| RK21_S85_L001_R1_001 | 1 | WOR5.16 | 5/20/2016 | 0 | 1 | 0.45427827 | 0.1581776 |  |  |  |  |  |  |  |
| RK33_S99_L001_R1_001 | 1 | WOR5.16 | 5/20/2016 | 0 | 2 | 0.23564106 | 0.04377693 |  |  |  |  | 12.9184851 |  |  |
| RK22_S86_L001_R1_001 | 1 | WOR5.16 | 5/20/2016 | 0 | 3 | 0.51393164 | 0.15104645 |  |  |  |  | 12.783331 |  |  |
|  |  | WOR5.16 | 5/27/2016 | 7 | 1 | 0.53097423 | 0.04214174 | 0.00918663 |  |  |  | 12.9719837 |  |  |

Table S1 Continued.

| ID | MiseqRun | Experiment | Date | Elapsed time | Replicate | H2 nM | H2 std | CH4 mM | CH4 STD | Isotope ch4 | Iso std | so4 mM | Cells ml | Std dev cells |
| --- | --- | --- | --- | --- | --- | --- | --- | --- | --- | --- | --- | --- | --- | --- |
|  |  | WOR5.16 | 5/27/2016 | 7 | 2 | 0.33184132 | 0.06559237 | 0.00238017 |  |  |  | 12.7326482 |  |  |
| RK6_S68_L001_R1_001 | 1 | WOR5.16 | 5/27/2016 | 7 | 3 | 0.22307574 | 0.06351896 | 0.00246369 |  |  |  | 13.1662678 |  |  |
| RK42_S22_L001_R1_001 | 1 | WOR5.16 | 6/3/2016 | 14 | 1 | 0.26750841 | 0.12173875 | 0.00233842 |  |  |  | 12.5468112 | 587849808 | 247704789 |
| RK43_S23_L001_R1_001 | 1 | WOR5.16 | 6/3/2016 | 14 | 2 | 0.37414223 | 0.08404409 | 0.00384168 |  |  |  | 12.4736027 | 449560920 | 199783971 |
| RK44_S24_L001_R1_001 | 1 | WOR5.16 | 6/3/2016 | 14 | 3 | 0.67701115 | 0.18632484 |  |  |  |  | 12.6003097 | 553005364 | 439186323 |
|  |  | WOR5.16 | 6/10/2016 | 21 | 1 | 0.26567935 | 0.04131197 | 0.00329884 |  |  |  | 9.56215684 |  |  |
|  |  | WOR5.16 | 6/10/2016 | 21 | 2 | 0.20513081 | 0.0498308 | 0.00384168 |  |  |  | 11.845699 |  |  |

Table S1 Continued.

| ID | MiseqRun | Experiment | Date | Elapsed time | Replicate | H2 nM | H2 std | CH4 mM | CH4 STD | Isotope ch4 | Iso std | so4 mM | Cells ml | Std dev cells |
| --- | --- | --- | --- | --- | --- | --- | --- | --- | --- | --- | --- | --- | --- | --- |

|  |  | WOR5.16 | 6/10/2016 | 21 | 3 | 0.10569562 | 0.01277318 | 0.00542847 |  |  |  | 4.8486555 |  |  |
| --- | --- | --- | --- | --- | --- | --- | --- | --- | --- | --- | --- | --- | --- | --- |
| RK3_S65_L001_R1_001 | 1 | WOR5.16 | 6/17/2016 | 28 | 1 | 0.20071523 | 0.03113766 | 0.00200436 |  |  |  | 11.9808532 | 870960920 | 238882160 |
| RK1_S63_L001_R1_001 | 1 | WOR5.16 | 6/17/2016 | 28 | 2 | 0.22489907 | 0.02542462 | 0.0019626 |  |  |  | 12.0822188 | 827405364 | 289361709 |
| RK4_S66_L001_R1_001 | 1 | WOR5.16 | 6/17/2016 | 28 | 3 | 0.27960607 | 0.06078822 | 0.00417574 |  |  |  | 11.6229762 | 1303249808 | 203096375 |
|  |  | WOR5.16 | 6/24/2016 | 35 | 1 |  |  | 0.00217139 |  |  |  | 11.1445868 | 174072031 | 63466824.4 |
|  |  | WOR5.16 | 6/24/2016 | 35 | 2 |  |  | 0.00238017 |  |  |  | 11.6823877 | 1218316475 | 200213272 |
|  |  | WOR5.16 | 6/24/2016 | 35 | 3 |  |  |  |  |  |  | 11.3163452 | 272072031 | 108082377 |

Table S1 Continued.

| ID | MiseqRun | Experiment | Date | Elapsed time | Replicate | H2 nM | H2 std | CH4 mM | CH4 STD | Isotope ch4 | Iso std | so4 mM | Cells ml | Std dev cells |
| --- | --- | --- | --- | --- | --- | --- | --- | --- | --- | --- | --- | --- | --- | --- |

| RK45_S25_L001_R1_001 | 1 | WOR5.16 | 7/1/2016 | 42 | 1 |  |  | 0.00250545 |  |  |  | 11.0488526 | 587849808 | 247704789 |
| --- | --- | --- | --- | --- | --- | --- | --- | --- | --- | --- | --- | --- | --- | --- |
| RK46_S26_L001_R1_001 | 1 | WOR5.16 | 7/1/2016 | 42 | 2 |  |  | 0.0025472 |  |  |  | 11.6091792 | 485494253 | 256501305 |
| RK47_S27_L001_R1_001 | 1 | WOR5.16 | 7/1/2016 | 42 | 3 |  |  | 0.00304829 |  |  |  | 11.2177953 | 1397983142 | 189496444 |
|  |  | WOR5.16 | 7/8/2016 | 49 | 1 |  |  | 0.00263072 |  |  |  | 10.9024356 | 1206338697 | 186363624 |
|  |  | WOR5.16 | 7/8/2016 | 49 | 2 |  |  |  |  |  |  | 10.5969309 |  |  |
|  |  | WOR5.16 | 7/8/2016 | 49 | 3 |  |  | 0.00204611 |  |  |  | 10.8573842 | 49938697.3 | 82432804.8 |
| RK2_S64_L001_R1_001 | 1 | WOR5.16 | 7/15/2016 | 56 | 1 |  |  | 0.00922839 |  |  |  | 10.0886949 | 280783142 | 104692790 |

Table S1 Continued.

| ID | MiseqRun | Experiment | Date | Elapsed time | Replicate | H2 nM | H2 std | CH4 mM | CH4 STD | Isotope ch4 | Iso std | so4 mM | Cells ml | Std dev cells |
| --- | --- | --- | --- | --- | --- | --- | --- | --- | --- | --- | --- | --- | --- | --- |

| RK7_S69_L001_R1_001 | 1 | WOR5.16 | 7/15/2016 | 56 | 2 |  |  | 0.00668119 |  |  |  | 11.0206955 | 233960920 | 99361046.8 |
| --- | --- | --- | --- | --- | --- | --- | --- | --- | --- | --- | --- | --- | --- | --- |
| RK13_76_L001_R1_001 | 1 | WOR5.16 | 7/15/2016 | 56 | 3 |  |  |  |  |  |  | 10.5279459 | 198027586 | 80657907.8 |
| RK17_S81_L001_R1_001 | 1 | WOR5.16 | 7/22/2016 | 63 | 1 |  |  | 0.00542847 |  |  |  | 10.6011544 | 253560920 | 105538771 |
| RK19_S83_L001_R1_001 | 1 | WOR5.16 | 7/22/2016 | 63 | 2 |  |  | 0.00279775 |  |  |  | 9.93664649 | 416894253 | 114887164 |
| RK23_S87_L001 | 1 | WOR5.16 | 7/22/2016 | 63 | 3 |  |  | 0.07098762 |  |  |  | 9.73954667 | 888383142 | 309705253 |

Table S1 Continued.

| ID | MiseqRun | Experiment | Date | Elapsed time | Replicate | H2 nM | H2 std | CH4 mM | CH4 STD | Isotope ch4 | Iso std | so4 mM | Cells ml | Std dev cells |
| --- | --- | --- | --- | --- | --- | --- | --- | --- | --- | --- | --- | --- | --- | --- |

| RK12_75_L001_R1_001 | 1 | WOR5.16 | 8/25/2016 | 97 | 1 | 0.61777811 | 0.11799971 |  |  |  |  | 8.76812614 | 465894253 | 111060040 |
| --- | --- | --- | --- | --- | --- | --- | --- | --- | --- | --- | --- | --- | --- | --- |
|  |  | WOR5.16 | 8/25/2016 | 97 | 2 | 0.81707026 | 0.1041026 | 0.00960421 |  |  |  | 9.88314797 | 242672031 | 72632319.9 |
| RK9_S72_L001_R1_001 | 1 | WOR5.16 | 8/25/2016 | 97 | 3 | 0.54692082 | 0.0837276 | 0.00751634 |  |  |  |  | 971138697 | 162028968 |
| RK20_S84_L001_R1_001 | 1 | WOR5.16 | 9/21/2016 | 124 | 1 | 0.64250852 | 0.27922589 |  |  |  |  | 6.66197381 | 1376205364 | 229218491 |
| RK34_S100_L001_R1_001 | 1 | WOR5.16 | 9/21/2016 | 124 | 2 | 0.2673946 | 0.01072335 |  |  |  |  | 7.03083204 | 1230294253 | 203804760 |

Table S1 Continued.

| ID | MiseqRun | Experiment | Date | Elapsed time | Replicate | H2 nM | H2 std | CH4 mM | CH4 STD | Isotope ch4 | Iso std | so4 mM | Cells ml | Std dev cells |
| --- | --- | --- | --- | --- | --- | --- | --- | --- | --- | --- | --- | --- | --- | --- |

| RK8_S70_L001_R1_001 | 1 | WOR5.16 | 9/21/2016 | 124 | 3 | 0.31304331 | 0.0315518 | 0.00396696 |  |  |  | 8.57665775 | 1144272031 | 206082012 |
| --- | --- | --- | --- | --- | --- | --- | --- | --- | --- | --- | --- | --- | --- | --- |
| RK16_S80_L001_R1_001 | 1 | WOR5.16 | 10/31/2016 | 164 | 1 | 0.08900689 | 0.08537794 |  |  |  |  |  |  |  |
| RK18_S82_L001_R1_001 | 1 | WOR5.16 | 10/31/2016 | 164 | 2 | 0.33161323 | 0.48886293 |  |  |  |  |  |  |  |
| RK32_S98_L001_R1_001 | 1 | WOR5.16 | 10/31/2016 | 164 | 3 | 0.08871519 | 0.06805387 |  |  |  |  |  | 290733333.3 | 109306746.1 |
| RK14_S77_L001 | 1 | WOR5.16 | 11/30/2016 | 194 | 1 | 0.2658185 | 0.10572547 |  |  |  |  |  |  |  |

Table S1 Continued.

| ID | MiseqRun | Experiment | Date | Elapsed time | Replicate | H2 nM | H2 std | CH4 mM | CH4 STD | Isotope ch4 | Iso std | so4 mM | Cells ml | Std dev cells |
| --- | --- | --- | --- | --- | --- | --- | --- | --- | --- | --- | --- | --- | --- | --- |

| RK10_S73_L001_R1_001 | 1 | WOR5.16 | 11/30/2016 | 194 | 2 | 0.39002857 | 0.1807838 |  |  |  |  |  |  |  |
| --- | --- | --- | --- | --- | --- | --- | --- | --- | --- | --- | --- | --- | --- | --- |
| RK5_S67_L001_R1_001 | 1 | WOR5.16 | 11/30/2016 | 194 | 3 | 0.22898279 | 0.04979109 |  |  |  |  | 4.49387583 | 286227586 | 109306746 |
|  |  | WOR5.16 | 1/6/2017 | 231 | 1 | 0.45348974 | 0.33842156 |  |  |  |  |  |  |  |
| RK15_S91_L001_R1_001 | 1 | WOR5.16 | 1/6/2017 | 231 | 2 | 0.16707859 | 0.09392149 | 0.00709876 |  |  |  |  | 596560920 | 96904993.6 |
| RK11_S74_L001_R1_001 | 1 | WOR5.16 | 1/6/2017 | 231 | 3 | 0.47191621 | 0.1269456 | 0.07098762 |  |  |  |  | 1008160920 | 176437544 |
|  |  | WOR5.16 | 3/28/2017 | 312 | 1 | 0.31311503 | 0.05217249 |  |  |  |  |  |  |  |

Table S1 Continued.

| ID | MiseqRun | Experiment | Date | Elapsed time | Replicate | H2 nM | H2 std | CH4 mM | CH4 STD | Isotope ch4 | Iso std | so4 mM | Cells ml | Std dev cells |
| --- | --- | --- | --- | --- | --- | --- | --- | --- | --- | --- | --- | --- | --- | --- |

| RK38_S17_L001_R1_001 | 1 | WOR5.16 | 3/28/2017 | 312 | 2 | 0.45070765 | 0.1468456 | 0.09186633 |  |  |  | 0.28973673 |  |  |
| --- | --- | --- | --- | --- | --- | --- | --- | --- | --- | --- | --- | --- | --- | --- |
|  |  | WOR5.16 | 3/28/2017 | 312 | 3 | 1.3544785 | 0.55048347 | 0.0405047 |  |  |  |  |  |  |
|  |  | WOR5.16 | 5/30/2017 | 375 | 1 | 0.21059075 | 0.09837163 |  |  |  |  |  |  |  |
| RK35_S101_L001_R1_001 | 1 | WOR5.16 | 5/30/2017 | 375 | 2 | 10.056337 | 0.36229294 | 0.33405939 |  |  |  |  | 659716475 | 125885715 |
|  |  | WOR5.16 | 5/30/2017 | 375 | 3 | 9.81260214 | 0.43110005 |  |  |  |  |  |  |  |
|  |  | WOR5.16 | 6/29/2017 | 405 | 1 |  |  |  |  |  |  |  |  |  |
|  |  | WOR5.16 | 6/29/2017 | 405 | 2 |  |  |  |  |  |  | 0.34914825 |  |  |
|  |  | WOR5.16 | 6/29/2017 | 405 | 3 |  |  |  |  |  |  |  |  |  |
| RK39_S18_L001_R1_001 | 1 | WOR5.16 | 9/19/2017 | 487 | 1 | 2.81170889 | 0.35681335 | 0.78503956 |  |  |  | 0.04223568 | 708716475 | 207859891 |

Table S1 Continued.

| ID | MiseqRun | Experiment | Date | Elapsed time | Replicate | H2 nM | H2 std | CH4 mM | CH4 STD | Isotope ch4 | Iso std | so4 mM | Cells ml | Std dev cells |
| --- | --- | --- | --- | --- | --- | --- | --- | --- | --- | --- | --- | --- | --- | --- |

| RK36_S15_L001_R1_001 | 1 | WOR5.16 | 9/19/2017 | 487 | 2 |  |  |  |  |  |  | 0.05603266 | 548649808 | 249510536 |
| --- | --- | --- | --- | --- | --- | --- | --- | --- | --- | --- | --- | --- | --- | --- |
| RK37_S16_L001_R1_001 | 1 | WOR5.16 | 9/19/2017 | 487 | 3 | 1.77557297 | 1.20731786 | 1.72875734 |  |  |  | 0.14078558 | 302560920 | 147742823 |
| RK30_S96_L001_R1_001 | 1 | WOR5.16 | 11/3/2017 | 532 | 1 | 0.48868623 | 0.21716683 |  |  |  |  | 1.22398986 | 894916475 | 194062970 |
| RK31_S97_L001_R1_001 | 1 | WOR5.16 | 11/3/2017 | 532 | 2 | 0.68988443 | 0.44438531 |  |  |  |  | 1.61903421 | 866605364 | 166862261 |
| RK29_S95_L001 | 1 | WOR5.16 | 11/3/2017 | 532 | 3 | 0.82375197 | 0.24318706 |  |  |  |  | 0.89539631 | 968960920 | 145180179 |

Table S1 Continued.

| ID | MiseqRun | Experiment | Date | Elapsed time | Replicate | H2 nM | H2 std | CH4 mM | CH4 STD | Isotope ch4 | Iso std | so4 mM | Cells ml | Std dev cells |
| --- | --- | --- | --- | --- | --- | --- | --- | --- | --- | --- | --- | --- | --- | --- |

|  |  | WOR5.16 | 12/11/2017 | 570 | 1 | 0.34162838 | 0.04873845 | 0.1085693 |  |  |  | 0 |  |  |
| --- | --- | --- | --- | --- | --- | --- | --- | --- | --- | --- | --- | --- | --- | --- |
| RK28_S94_L001_R1_001 | 1 | WOR5.16 | 11-Dec | 570 | 2 | 0.19708483 | 0.04143012 | 0.05929554 |  |  |  | 0.18020555 | 1000538697 | 201678222 |
| RK27_S93_L001_R1_001 | 1 | WOR5.16 | 11-Dec | 570 | 3 | 0.64660562 | 0.11975332 | 0.09520693 |  |  |  | 1.03055047 | 1203072031 | 269032237 |
|  |  | WOR5.16 | 1/27/2018 | 617 | 1 | 0.78328681 | 0.28457135 | 0.07349307 |  |  |  |  |  |  |
| RK25_S89_L001_R1_001 | 1 | WOR5.16 | 1/27/2018 | 617 | 2 | 1.27511282 | 1.2669162 | 0.05762524 |  |  |  | 1.21357173 | 1201983142 | 222208416 |
| RK26_S92_L001_R1_001 | 1 | WOR5.16 | 1/27/2018 | 617 | 3 | 1.68403552 | 0.97730234 |  |  |  |  | 0.47585527 | 950449808 | 216292386 |

Table S1 Continued.

| ID | MiseqRun | Experiment | Date | Elapsed time | Replicate | H2 nM | H2 std | CH4 mM | CH4 STD | Isotope ch4 | Iso std | so4 mM | Cells ml | Std dev cells |
| --- | --- | --- | --- | --- | --- | --- | --- | --- | --- | --- | --- | --- | --- | --- |

| RK40_S19_L001_R1_001 | 1 | WOR5.16 | 3/1/2018 | 650 | 2 | 2.01935948 | 1.08116423 | 0.03312756 | 2.4109E-06 |  |  |  | 1206338697 | 237264418 |
| --- | --- | --- | --- | --- | --- | --- | --- | --- | --- | --- | --- | --- | --- | --- |
| RK41_S20_L001_R1_001 | 1 | WOR5.16 | 3/1/2018 | 650 | 3 | 0.77804779 | 0.19279874 | 0.15018753 | 0.00173793 |  |  |  | 1155160920 | 301812545 |
| RK24_S88_L001_R1_001 | 1 | WOR5.16 | 3/1/2018 | 650 | 1 | 1.03551141 | 0.29596793 | 0.09743399 | 2.4109E-05 |  |  | 0.49274954 | 998360920 | 239919664 |
|  |  | WOR5.16 | 4/11/2018 | 691 | 1 | 0.31544763 | 0.03121203 | 0.03368432 | 0.00039909 |  |  |  |  |  |
|  |  | WOR5.16 | 4/11/2018 | 691 | 2 | 1.25305787 | 1.33123056 | 0.00346587 | 3.3083E-05 |  |  |  |  |  |
| RK48_S28_L001_R1_001 | 1 | WOR5.16 | 4/11/2018 | 691 | 3 | 0.60487614 | 0.16382067 | 0.23648621 | 0.00113474 |  |  | 0.1210756 |  |  |

Table S1 Continued.

| ID | MiseqRun | Experiment | Date | Elapsed time | Replicate | H2 nM | H2 std | CH4 mM | CH4 STD | Isotope ch4 | Iso std | so4 mM | Cells ml | Std dev cells |
| --- | --- | --- | --- | --- | --- | --- | --- | --- | --- | --- | --- | --- | --- | --- |

| WOR_5_24_18_B1 | 2 | WOR5.16 | 5/23/2018 | 733 | 1 | 0.52200057 | 0.10024004 | 0.09047442 | 4.8217E-05 |  |  | 0.6757708 | 880760920 | 142093202 |
| --- | --- | --- | --- | --- | --- | --- | --- | --- | --- | --- | --- | --- | --- | --- |
| WOR_5_24_18_B2 | 2 | WOR5.16 | 5/23/2018 | 733 | 2 | 0.61711295 | 0.2475372 | 0.01816448 | 2.3373E-05 |  |  | 0.09854991 | 1049538697 | 222704652 |
| WOR_5_24_18_B3 | 2 | WOR5.16 | 5/23/2018 | 733 | 3 | 0.82824597 | 0.18391694 | 0.0427596 | 0.00021376 |  |  | 0.18302126 | 690205364 | 137241118 |
| WOR_6_28_18_B1 | 2 | WOR5.16 | 6/28/2018 | 769 | 1 | 0.63570104 | 0.18930431 | 0.06681188 | 8.3515E-05 |  |  | 1.46698578 | 569338697 | 144235087 |
| WOR_6_28_18_B2 | 2 | WOR5.16 | 6/28/2018 | 769 | 2 | 1.74997466 | 0.69640339 | 0.0064028 | 1.2757E-05 |  |  | 2.66647895 | 612894253 | 132164689 |
| WOR_6_28_18_B3 | 2 | WOR5.16 | 6/28/2018 | 769 | 3 | 3.22761695 | 2.41453197 |  |  |  |  | 0.04223568 | 831760920 | 152401527 |
| WOR_8_1 | 2 | WOR5.16 | 8/1/2018 | 802 | 1 | 0.47215626 | 0.21531593 | 0.08629868 | 0.0003789 |  |  |  |  |  |

Table S1 Continued.

| ID | MiseqRun | Experiment | Date | Elapsed time | Replicate | H2 nM | H2 std | CH4 mM | CH4 STD | Isotope ch4 | Iso std | so4 mM | Cells ml | Std dev cells |
| --- | --- | --- | --- | --- | --- | --- | --- | --- | --- | --- | --- | --- | --- | --- |

| WOR_8_1_18_B2 | 2 | WOR5.16 | 8/1/2018 | 802 | 2 | 0.31524201 | 0.08949674 | 0.02037762 | 0.00012842 |  |  |  | 274249808 | 159972003 |
| --- | --- | --- | --- | --- | --- | --- | --- | --- | --- | --- | --- | --- | --- | --- |
| WOR_8_1_18_B3 | 2 | WOR5.16 | 8/1/2018 | 802 | 3 | 1.28525899 | 0.11883562 | 0.00883865 | 2.4468E-05 |  |  | 2.4130649 | 826316475 | 156941523 |
| WOR_11_2_18_B1 | 2 | WOR5.16 | 11/1/2018 | 895 | 1 | 0.20049527 | 0.02025325 | 0.21727779 | 0.00297455 |  |  | 6.9491764 | 194911111 | 33756161 |
| WOR_11_2_18_B2 | 2 | WOR5.16 | 11/1/2018 | 895 | 2 | 0.16106491 | 0.05267123 | 0.01279169 | 0.0001869 |  |  | 13.2591863 | 193822222 | 32021991 |
| WOR_11_2_18_B3 | 2 | WOR5.16 | 11/1/2018 | 895 | 3 | 0.23419407 | 0.02075477 | 0.00044541 | 7.7148E-06 |  |  | 2.21033366 |  |  |

Table S2. Concentration of total RNA, final library concentration and average library size for WOR 5.16 transcriptomes.

| Days Elapsed | RNA Concentration (ng/uL) | Library Concentration (ng/uL) | Avg Library size (bp) |
| --- | --- | --- | --- |
| 650 | 15.8 | 41.40 | 426 |
| 532 | 23.4 | 41.20 | 413 |
| 487 | 18.1 | 43.20 | 411 |
| 375 | 7.94 | 39.00 | 407 |
| 312 | 18.2 | 39.60 | 394 |
| 231 | 22.8 | 38.40 | 414 |
| 194 | 13.4 | 40.40 | 441 |
| 124 | 22.0 | 43.20 | 429 |
| 63 | 20.2 | 39.60 | 442 |
| 0 | 16.4 | 39.80 | 435 |
